# Chemical Characterization and *in Vitro* Antibacterial Activity of *Myrcianthes hallii* (O. Berg) McVaugh (Myrtaceae), a Traditional Plant Growing in Ecuador

**DOI:** 10.3390/ma9060454

**Published:** 2016-06-07

**Authors:** Patricia Chavez Carvajal, Erika Coppo, Arianna Di Lorenzo, Davide Gozzini, Francesco Bracco, Giuseppe Zanoni, Seyed Mohammad Nabavi, Anna Marchese, Carla Renata Arciola, Maria Daglia

**Affiliations:** 1 Department of Drug Sciences, Medicinal Chemistry and Pharmaceutical Technology Section, University of Pavia, Via Taramelli 12, 27100 Pavia, Italy; patylu_170787@hotmail.com (P.C.C.); arianna.dilorenzo01@universitadipavia.it (A.D.L.); 2 Microbiology Unit, DISC, University of Genoa, IRCCS-San Martino IST, Largo Rosanna Benzi 10, 16132 Genoa, Italy; erika.coppo@unige.it (E.C.); anna.marchese@unige.it (A.M.); 3 Department of Chemistry, University of Pavia, Via Taramelli 10, 27100 Pavia, Italy; davide.gozzini@unipv.it (D.G.); gz@unipv.it (G.Z.); 4 Department of Earth and Environmental Sciences, University of Pavia, Via Sant’Epifanio 14, 27100 Pavia, Italy; francesco.bracco@unipv.it; 5 Applied Biotechnology Research Center, Baqiyatallah University of Medical Sciences, Tehran 19395-5487, Iran; nabavi208@gmail.com; 6 Research Unit on Implant Infections, Rizzoli Orthopaedic Institute, via di Barbiano 1/10, 40136 Bologna, Italy; 7 Department of Experimental, Diagnostic and Specialty Medicine (DIMES), University of Bologna, Via San Giacomo 14, 40126 Bologna, Italy

**Keywords:** *Myrcianthes hallii*, acidic hydro-methanolic leaf extract, phytochemical composition, antibacterial activity, multidrug-resistant bacteria, polyphenols, anti-infective biomaterials

## Abstract

*Myrcianthes hallii* (O. Berg) McVaugh (Myrtaceae) is a plant native to Ecuador, traditionally used for its antiseptic properties. The composition of the hydro-methanolic extract of this plant was determined by submitting it to ultra-high performance liquid chromatography (UHPLC) hyphenated to heated-electrospray ionization mass spectrometry and UV detection. The presence of antimicrobial components prompted us to test the extract against methicillin-resistant and methicillin-susceptible *Staphylococcus aureus*, multidrug-resistant and susceptible *Escherichia coli*, *Pseudomonas aeruginosa*, *Enterococcus* spp. and *Streptococcus pyogenes* strains. The chromatographic analysis led to the identification of 38 compounds, including polyphenols and organic acids, and represents the first chemical characterization of this plant. The extract showed modest antibacterial activity against all tested bacteria, with the exception of *E. coli* which was found to be less sensitive. Whilst methicillin-resistant strains usually display resistance to several drugs, no relevant differences were observed between methicillin-susceptible and resistant strains. Considering its long-standing use in folk medicine, which suggests the relative safety of the plant, and the presence of many known antibacterial polyphenolic compounds responsible for its antibacterial activity, the results show that *M. hallii* extract could be used as a potential new antiseptic agent. Moreover, new anti-infective biomaterials and nanomaterials could be designed through the incorporation of *M. hallii* polyphenols. This prospective biomedical application is also discussed.

## 1. Introduction

The Myrtaceae family includes about 30 genera and 1500 species found in the Neotropics. In Ecuador there are 15 native genera and about 200 species. Native species are mainly grown for their edible fruits and wood and some are used as medicinal plants due to their biological properties [1]. In recent years, increasing interest in herbal medicine has been registered in developed and developing countries, and much attention has been paid to natural antibacterial substances for use in alternative therapies against conventionally resistant infections or as new antiseptic agents. In the last decade, many species of Myrtaceae (including those from the *Myrtus*, *Eucalyptus*, *Psidium*, and *Syzygium* genera) have been studied for their antimicrobial properties [2,3,4,5]. *Myrcianthes* is a genus of Myrtaceae that includes shrubs and small trees. At present, 38 species of *Myrcianthes* are known to be distributed across several countries of Central and South America, from Mexico to Chile, including Ecuador [6]. Although *Myrcianthes* is closely related to the large genus *Eugenia* L. recent studies have confirmed its individuality and role as a sister group to the rest of the *Eugenia* clades [7]. Thanks to recent taxonomic research [8,9], new species belonging to the *Myrcianthes* genus have been described. *Myrcianthes hallii* (O. Berg) McVaugh is a medicinal and aromatic species commonly known in Ecuador as “arrayán” [10,11]. It grows as a shrub or a tree up to 8 m high. Leaves and branchlets are nearly glabrous, flowers are tetramerous and the hypanthium is characteristically densely pale and strigose. This species is recorded in Peru, Ecuador, Venezuela and possibly in Colombia [12,13]. In Ecuador *M. hallii* is found both native and cultivated, growing in the Andean region from 2500 to 3000 metres above mean sea level (MAMSL), and mainly in the provinces of Azuay, Bolivar, Carchi, Chimborazo, Imbabura, Loja and Pichincha [11,13]. Its leaves and berries are used in both traditional medicine and in cosmetics and foods, as a culinary herb or spice. In traditional medicine, arrayán is consumed as an infusion and a decoction for its antiseptic, haemostatic, and balsamic properties. Moreover, dried ground leaves applied to wounds aid healing and are used in baths, vapors and massages for their stimulating and tonic properties. Green leaves are used to clean teeth and gums, with the added effect of whitening teeth naturally. Finally, fresh leaves macerated in olive oil seem to prevent hair loss [10,11,14]. The chemical compositions of plants belonging to the Myrtaceae family (such as *Eucalyptus* and *Myrtus*) have been widely studied. *Eucalyptus* species are rich sources of biologically active terpenoids, tannins, flavonoids and phloroglucinol derivatives [3,15,16]. In *Myrtus communis* L. (myrtle) the presence of different polyphenolic classes is reported, including phenolic acids, flavonoids, galloyl derivatives and hydrolysable tannins [2,17,18]. Some of the main polyphenols found in myrtle are also present in green tea (catechin and gallocatechin derivatives) which is widely recognized as an excellent source of powerful nutraceuticals [17,18]. A recent study on leaf extract obtained from *Myrcianthes cisplatensis* (Cambess.) O. Berg, reported the presence of α-methyl-1-(2′,4′,6′-trimethoxyphenyl)-1-propanone, known as conglomerone, which shows antibacterial activity against methicillin-sensitive and resistant *Staphylococcus aureus* strains [19].

Although studies reporting the composition of essential oils or the biological activity of extracts do exist for several species of *Myrcianthes* (*M. pungens* (O. Berg) D. Legrand, *M. cisplatensis* (Cambess.) O. Berg, *M. pseudomato* (D. Legrand) McVaugh, *M. fragrans* (Sw.) McVaugh, *M. rhopaloides* (Kunth) McVaugh, *M. osteomeloides* (Rusby) McVaugh, and *M. coquimbensis* (Barnéoud) Landrum et Grifo) [19,20,21,22,23,24,25,26], the available information concerning *M. hallii* is particularly limited. Therefore, in view of the fact that *M. hallii* is commonly employed as an antibacterial agent in Ecuadorian folk medicine, and therefore is likely safe and effective, and the importance of finding new anti-infective extracts to be loaded into or coated onto biomaterials, the aims of this study were to investigate the chemical composition of *M. hallii* and its antibacterial activity against Gram positive and Gram negative multidrug-resistant strains.

## 2. Results

### 2.1. UHPLC-PDA-hESI-MSn Analysis of LMW Fraction of MHE

**Figure 1:**
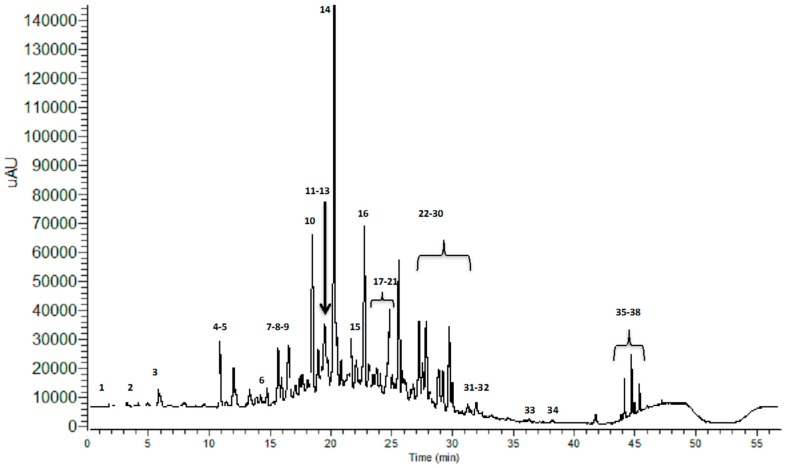
UHPLC-UV chromatographic profile of the LMW fraction of MHE, registered at 280 nm.

The acidic hydro-methanolic leaf extract of *M. hallii* (MHE) was submitted to dialysis, using a membrane with a nominal molecular weight cut-off (MWCO) of 3500 Da. A low molecular weight (LMW) fraction, suitable for UHPLC-PDA-hESI-MSn analysis, was obtained. The chromatographic profile, acquired at 280 nm, is reported in Figure 1. Thirty-eight compounds were identified based on their chromatographic behaviour and mass spectra, in comparison with the literature. Table 1 summarizes the identified compounds, their retention times and *m*/*z* values for the parent ion and fragment ions.

The analysis shows the presence of 29 flavonoids, consisting of (a) 5 flavan-3-ols (gallocatechin, catechin, epigallocatechin, epicatechin and epigallocatechin gallate); (b) 7 condensed tannin derivatives (three isomers of procyanidin dimer, procyanidin-gallate, and three isomers of procyanidin-digallate); (c) 12 flavonols (myricetin 3-*O*-galactoside/myricetin 3-*O*-glucoside, myricetin 3-*O*-arabinoside, myricetin 3-*O*-rhamnoside, quercetin, quercetin hexosyl-gallate, quercetin 3-*O*-rhamnoside, quercetin 3-*O*-galactoside/quercetin 3-*O*-glucoside, quercetin 3-*O*-arabinose, acylated myricetrin, kaempferol 3-*O*-glucoside, aromadendrin-rhamnoside, and cypellogin A or B); (d) a flavanone derivative (pinobanksin 3-*O*-butyrate); (e) a flavone derivative (apigenin-hexoside) and (f) 3 anthocyanin derivatives (cyanidin-dihexoside, cyanidin-3-*O*-rutinoside, and cyanidin-3-glucoside/cyaniding-3-galactoside). Moreover, in MHE a phenolic acid (gallic acid), 5 hydrolysable tannins (hexahydroxydiphenoyl-glucose, hexahydroxydiphenoyl-galloylglucose, digalloylglucose, trigalloylglucose, and monogalloyl-quinic acid), and 3 organic acids (quinic acid, malic acid and gluconic acid) were detected.

In further detail, five flavan-3-ols were identified in MHE. Peaks **9** and **13** were identified as gallocatechin and epigallocatechin, respectively, by comparing their MS^2^ spectra with literature data (MW 306). Their parent ions (*m*/*z* 305) produced characteristic fragment ions at *m*/*z* 261, 221, 219, 179, 167, and 165, generated by the cleavage of the A ring, the heterocyclic ring fission, and the retro-Diels-Alder fission occurring during the fragmentation process as reported by Dou *et al.* [27]. It was possible to identify the epimers by comparison with commercial standards. Peaks **15** and **18**, which both showed a parental ion at *m*/*z* 289, were assigned to catechin and epicatechin respectively (MW 290). Their MS^2^ spectra showed the presence of ions at *m*/*z* 245, 205, 203, and 137, generated by the cleavage of ring A and the retro-Diels-Alder fission occurring during the fragmentation process [27]. It was again possible to identify the epimers by comparison with commercial standards. Peak **19**, which produced a molecular ion [M–H]^−^ at *m*/*z* 457, was identified as epigallocatechin-3-gallate (MW 458) since it produced fragment ions at *m*/*z* 305 and 169 corresponding to the deprotonated ion of epigallocatechin and gallic acid [27]. It was possible to identify this compound by comparison with the commercial standard.

Twelve flavonols were identified in MHE: four myricetin derivatives, five quercetin derivatives, a quercetin aglycone, and two kaempferol derivatives. Four myricetin derivatives were identified in MHE due to their MS and MS/MS spectra. Peaks **21**, **23**, **24**, **32** were assigned to myricetin derivatives due to the presence of the aglycone at *m*/*z* 317, in their MS^2^ spectra. Peak **21** was identified as myricetin glucoside or galactoside (MW 480) due to the parental ion at *m*/*z* 479 producing the MS^2^ fragment [M–162]^−^; peak **24** was assigned to myricetin rhamnoside (MW 464) due to the loss of 146 Da from the molecular ion at *m*/*z* 463, according to Faria *et al.* [28]. Peak **23** was identified as myricetin arabinoside (MW 450), since it produced the MS^2^ ion at *m*/*z* 317 from the molecular ion at *m*/*z* 449, due to the loss of [M–132]^−^ corresponding to the arabinoil moiety. Peak **32** was assigned to acylated myricitrin (MW 506). Its molecular ion at *m*/*z* 505 produced a base peak at *m*/*z* 316, corresponding to the myricetin aglycone, and a fragment at *m*/*z* 463, due to the loss of the acylic moiety. In MHE five quercetin derivatives were identified, because all peaks presented the characteristic fragment ions of quercetin aglycone (e.g., MS^n^ data at *m*/*z* 300, 301, 179). Peaks **25**, **27**, **30** were assigned to quercetin glucoside or galactoside (MW 464), quercetin arabinoside (MW 434), and quercetin rhamnoside (MW 448), since they yielded the fragments [M–162]^−^, [M–132]^−^, [M–146]^−^, respectively. Peak **22** was identified as quercetin hexosylgallate (MW 616), since the molecular ion [M–H]^−^ at *m*/*z* 615 produced a fragment at *m*/*z* 463, corresponding to the loss of a galloyl group, and a fragment at *m*/*z* 301 due to the loss of a hexosyl group, corresponding to the aglycone [15]. Peak **34** may correspond to Cypellogin A or B (MW 630), which is a quercetin glucoside or galactoside acylated with an oleuropeic acid residue [29]; in fact, the compound yielded a base peak at *m*/*z* 301 corresponding to the aglycone. Quercetin (MW 302) was identified as peak **35** due to the presence of the molecular ion [M–H]^−^ at *m*/*z* 301. With regards to kaempferol derivatives, peak **28** was assigned to kaempferol 3-*O*-glucoside (MW 448) by observing the MS^2^ data closely related to the glycosylation position. According to Ablajan *et al.* [30], peak **28** showed a MS^2^ spectrum typical of a 3-*O*-glucosyl derivative. It produced a base peak at *m*/*z* 284, related to the homolytic cleavage of deprotonated flavonoid glycosides, a fragment at *m*/*z* 255 more abundant than that registered at *m*/*z* 257 and a fragment at *m*/*z* 327 that is not present in 7-*O*-glucosyl derivatives. Peak **31** was identified as aromadendrin rhamnoside (MW 434), since the molecular ion [M–H]^−^ at *m*/*z* 433 produced the fragment [M–146]^−^ corresponding to the aglycon at *m*/*z* 287.

Regarding flavanone and flavone derivatives, peak **38** was assigned to pinobanksin 3-*O*-butyrate, the parent ion (*m*/*z* 343) producing fragment ions as reported by Chua *et al.* [31]. Peak **37** was identified as apigenin-hexoside, since it produced a molecular ion [M–H]^−^ at *m*/*z* 431, and yielded a fragment ion at *m*/*z* 269 corresponding to apigenin aglycone due to the loss of a hexosyl group. Three anthocyanidin derivatives were identified in MHE. Peaks **8**, **10**, **29** showed the presence of the cyanidin aglycone at *m*/*z* 287 in their MS^2^ spectra. Peak **8** was assigned to cyanidin-dihexoside (MW 610), since the parent ion (*m*/*z* 611) produced fragment ions at *m*/*z* 449 and *m*/*z* 287, corresponding to the loss of the sugar molecules linked to the aglycone. Peak **10** was identified as cyanidin-3-rutinoside thanks to the pseudomolecular ion [M + H]^+^ at *m*/*z* 595 and its MS^2^ fragments at *m*/*z* 433, caused by the loss of one of the sugar molecules linked to the aglycone, and *m*/*z* 287, corresponding to the cyaniding aglycone (MW 594). Peak **29**, which produced a molecular ion [M + H]^+^ at *m*/*z* 449 and yielded a fragment ion at *m*/*z* 287 corresponding to the loss of the hexosyl moiety on MS^2^, may be assigned to the glucoside or galactoside derivative of cyanidin (MW 448).

With regards to benzoic acids, gallic acid (MW 170) was identified as peak **4** in MHE, since it produced a base peak at *m*/*z* 125 corresponding to the loss of a carboxyl group [M–H–CO_2_]^−^.

As far as tannins are concerned, condensed tannins and hydrolizable tannins (gallotannins and ellagitanninis) were detected. Seven condensed tannin compounds were detected in MHE. Three peaks (**12**, **14**, **16**) showed molecular ions at *m*/*z* 577 with the same MS^2^ fragmentation pattern (*m*/*z* 425, 407, 289), but different retention times: consequently they can be considered to be three isomers of procyanidin (MW 578) [32]. Peak **20** produced a molecular ion [M–H]^−^ at *m*/*z* 729 and the most significant MS^2^ fragments at *m*/*z* 577, corresponding to the loss of a galloyl group, and at *m*/*z* 289, corresponding to the monomer: for these reasons peak **20** was assigned to a monogalloyl procyanidin dimer. Peaks **7**, **11**, **17** produced a molecular ion [M–H]^−^ at *m*/*z* 881, which could correspond to procyanidin digallate, and the same MS^2^ fragmentation spectra. The main fragments produced were at *m*/*z* 729 (corresponding to the loss of a galloyl group), at *m*/*z* 711 (corresponding to the loss of a molecule of gallic acid), at *m*/*z* 577 (corresponding to procyanidin), and at *m*/*z* 289 (corresponding to the monomer (−)-epicatechin or (−)-catechin). Consequently, peaks **7**, **11**, and **17** were assigned to three different isomers of procyanidin digallate. With regards to hydrolizable tannins, peaks **3** and **5** were assigned to hesahydroxydiphenoyl-glucose and hesahydroxydiphenoyl-galloylglucose, respectively. Peak **3** was identified as hesahydroxydiphenoyl-glucose (MW 480), since it produced a molecular ion at *m*/*z* 481 and a MS^2^ fragment ion at *m*/*z* 301, corresponding to the loss of a glucose unit as reported in the literature [33]. Peak **5** produced a molecular ion at *m*/*z* 633, an intense fragment ion at *m*/*z* 301 suggesting the loss of a galloylglucose unit, and a fragment ion at *m*/*z* 481, corresponding to the molecular ion of hesahydroxydiphenoyl-glucose, due to the loss of a galloyl unit. Through comparison with data from the literature [33], the fragmentation pattern of this molecule suggests that the galloyl moiety is directly linked to the glucose unit, so the compound was identified as hesahydroxydiphenoyl-galloylglucose (MW 634). Peak **33** was assigned to digalloylglucose (MW 484), since it provided the typical MS^2^ fragment ions at *m*/*z* 331 [M–H–152]^−^ and *m*/*z* 169 [M–H–162]^−^, corresponding to the sequential loss of the galloyl and the glucosyl moiety, respectively [33]. Moreover, peak **36** was identified as trigalloylglucose (MW 636), since the molecular ion at *m*/*z* 635 produced a base peak at *m*/*z* 483, due to the loss of a galloyl group, leading to *m*/*z* typical of digalloyl glucose, and a fragment at *m*/*z* 465 due to the loss of gallic acid. Finally, peak **6** was assigned to monogalloyl quinic acid (MW 344). It showed a molecular ion at *m*/*z* 343 and a MS^2^ spectrum characterized by the presence of fragment ions at *m*/*z* 169 and 125. The neutral loss of 174 Da corresponds to quinic acid. 

Finally, some organic acids (quinic acid, malic acid and gluconic acid) were identified in MHE. Peak **1** was identified as quinic acid (MW 192), with a retention time of approximately 2 min and MS^2^ fragments at *m*/*z* 173 [M–H–H_2_O]^−^, corresponding to the loss of a water molecule, and at *m*/*z* 127 [M–H–CO–2H_2_O]^−^, corresponding to the loss of the carboxylic moiety and two water molecules. Peak **2**, showing a retention time of 3.10 min and a MS^2^ fragment at *m*/*z* 115 [M–H–H_2_O]^−^, was identified as malic acid. Peak **26** yielded to MS^2^ fragments at *m*/*z* 179 [M–H–H_2_O]^−^, due to the loss of a water molecule, and at *m*/*z* 135 [M–H–CO–H_2_O]^−^ representing the 60% of the base peak, due to the loss of the carboxylic group and a water molecule. Thus, peak **26** was identified as gluconic acid (MW 198) [34].

### 2.2. Antibacterial Activity of MHE

**Figure 2:**
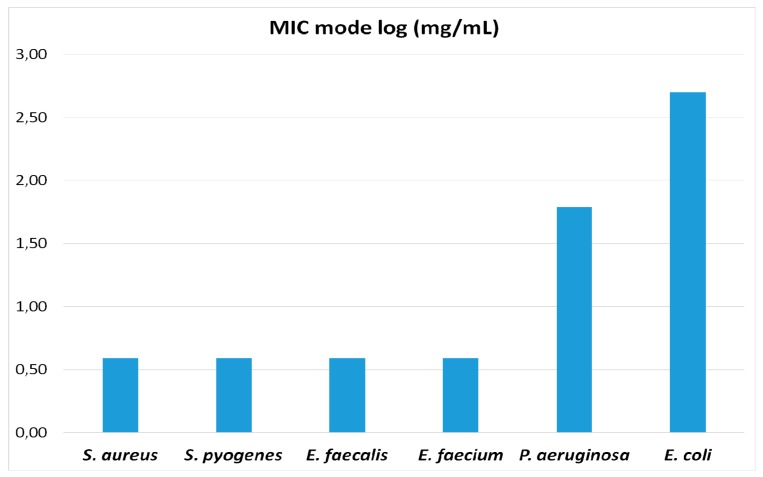
MIC values within groups follow a modal distribution. MIC mode values (expressed as log_10_) for the different bacterial species assayed are represented in the figure. The four Gram-positive species (*S. aureus*, *S. pyogenes*, *E. faecalis*, *E. faecium*) exhibit equal values of MIC mode, while the MIC mode values against the two Gram-negative species (*P. aeruginosa* and *E. coli*) are much higher (*E. coli* > *P. aeruginosa*).

The antibacterial activity of MHE obtained from *M. hallii* dried leaves was tested against ten MRSA and MSSA, ten *E. coli*, ten *P. aeruginosa*, ten *Enterococcus* spp, and ten *S. pyogenes* strains. MHE showed antibacterial activity against all the tested *S. aureus* (MIC range 0.007–0.0019 gr/mL), *P. aeruginosa* (MIC range 0.125–0.062 gr/mL), *S*. *pyogenes* (MIC range 0.007–0.0039 gr/mL) and *Enterococcus* spp. (MIC range 0.0039–0.0019 gr/mL). MHE was found to be less active against *E. coli* strains (MIC range 0.25 > 0.5 gr/mL) (Table 2 and Table 3 and Figure 2). Our data showed that MHE extract can inhibit bacterial strains irrespectively of their mechanisms of resistance. No significant differences were observed between strains carrying well-known mechanisms of resistance and susceptible ones, because one-dilution differences in this kind of analysis are taken for granted.

## 3. Discussion

This research represents the first report on the isolation and identification of polyphenols and organic acids from *M. hallii* to date. UHPLC-PDA-hESI-MSn analysis revealed the presence of thirty-eight compounds, belonging to flavonoids, phenolic acids, tannins and organic acids. Some of them have been previously identified in extracts obtained from plants and fruits belonging to the Myrtaceae family, whilst for others, this is the first report of their identification in a Myrtaceae species.

In MHE extract, 29 flavonoids were identified, consisting of flavan-3-ols, condensed tannin derivatives, flavonols, a flavanone and a flavone derivative and anthocyanin derivatives. Flavan-3-ols were also detected in the methanolic extract of the bark of *Tristaniopsis callobuxus* Brongn. & Gris, by Bellosta *et al.* [35], which showed the presence of gallocatechin and epigallocatechin. Catechin was also detected in the methanolic extracts obtained from the air-dried leaves of *Myrtus communis* L. (myrtle) and *Eucalyptus globulus* Labill [16,18]. Amongst Myrtaceae, flavonol derivatives were also detected in the methanolic extracts of *Eucalyptus globulus*. For myrtle, infusions and leaf methanolic extracts were both shown to contain myricetin and quercetin derivatives [2,18]. In the methanolic extract obtained from the fruits of *Myrciaria vexator* McVaugh, Dastmalchi *et al.* identified the following flavonols, quercitrin (quercetin 3-rhamnoside), quercetin 3-glucoside, and myricetin [36]. Moreover, in the fruits of *Myrcianthes pungens* (O. Berg) D. Legrande, De Mello Andrade *et al.* showed the presence of quercitrin [24]. Regarding anthocyanins, in the fruits of *Myrciaria dubia* (Kunth) McVaugh, a plant native of Amazonian rainforest, cyanidin-3-glucoside was identified as the main pigment [37]. The other two cyanidin-derivatives identified in MHE have never been reported in plants belonging to Myrtaceae family. Condensed tannins, such as procyanidin, were detected in many species belonging to *Eucalyptus* and *Eugenia* genera [38]. Ellagitannis have only been found in dicotyledoneous angiosperms and Myrtaceae are indicated as being rich in ellagitannins. In support of this fact, ellagitannis were detected in *Callistemon lanceolatus* Sweet, *Eucalyptus alba* Reinw. Ex Blume, *Eugenia grandis* Wight, *Kunzea ambigua* (Sm.) Druce, *Melaleuca squarrosa* Sm., *Pimenta dioica* (L.) Merr., *Siphoneugena densiflora*, *Syzygium aqueum* (Burm. f.) Alston, and *Syzygium aromaticum* (L.) Merr & L.M. Perry [39]. In MHE, gallic acid, hydrolysable tannins and organic acids have been detected. Gallic acid is a secondary metabolite of plants, mainly formed from 3-dehydroshikimic acid through the shikimic acid pathway [40]. It is widely distributed in many different families of higher plants, both in the free state and as a part of more complex molecules such as ester derivatives or polymers. Amongst Myrtaceae, gallic acid was detected in the methanolic extract obtained from the leaves of *Eucalyptus globulus* [16], the fruits of *Rhodomyrtus tomentosa* (Aiton) Hassk. [41], the methanolic extract of the bark of *Tristaniopsis callobuxus* [35], leaf infusions or methanolic extract obtained from *Myrtus communis* [2,18] and the seed and fruit extracts of *Syzygium cumini* (L.) Skeels [42]. Quinic acid has been identified in many plants in the Myrtaceae family, such as *Syzygium cumini* and *Myrtus communis* [17,43].

Amongst the thirty-eight compounds occurring in *M. hallii* leaf extract, many of them possess well-documented antibacterial activity [44]. For example, the *in vitro* antibacterial activity of catechin derivatives has been known since the 1990s and has been demonstrated against different strains, such as *Streptococcus mutans*, *E. coli*, *Clostridium perfringes* and *Bacillus cereus* [45,46,47]. Moreover, flavonols, especially myricetin, quercetin and kaempferol derivatives, are characterized by a remarkable antibacterial activity against both Gram-positive and Gram-negative bacteria, such as *S. aureus*, *Lactobacillus acidophilus*, *Porphyromonas gingivalis* and *Prevotella melaninogenica* [48]. Other categories of compounds with known antibacterial activity are gallotannins and procyanidins. The former showed antibacterial activity against Gram-positive food-borne bacteria (*i.e.*, *Clostridium botulinum*, *Bacillus subtilis*, *B. cereus*) [49]; the latter, especially those derived from berries, were found to be active against *E. coli*, *S. mutans* and oxacillin-resistant *S. aureus* [50]. On this basis, we evaluated MHE activity against different strains of *S. aureus*, *P. aeruginosa*, *S. pyogenes*, *Enterococcus* spp. and *E. coli.* To the best of our knowledge, no data are available on the antibacterial activity of *M. hallii* against drug resistant bacteria. Although MHE was found to be as rich in polyphenolic components that could exert antibacterial activity, our results showed that the antibacterial activity of MHE against *S. aureus*, *P. aeruginosa*, *S. pyogenes* and *Enterococcus* spp. strains is modest but appreciable, especially against *Enterococcus* spp. MHE was shown to be much less active against *E. coli* strains. Moreover, our data shows that, whilst methicillin-resistant strains usually display resistance to several drugs, no relevant differences were observed between methicillin-susceptible and resistant strains. Our results agree with earlier studies carried out on other species belonging to the *Myrcianthes* genus. The ethanol extract obtained from *Myrcianthes discolor* (Kunth) McVaugh dried leaves showed antibacterial activity against a *S. aureus* strain isolated from laryngitis samples, but did not show any activity against two *E. coli* strains isolated from urinary tract infection samples [51,52]. Moreover, *M. cisplatensis* showed antibacterial activity against methicillin-sensitive and resistant *S. aureus* strains [19]. According to the results obtained from UHPLC-PDA-hESI-MSn analysis, the appreciable antibacterial activity of MHE against *S. aureus*, *P. aeruginosa*, *S. pyogenes*, and especially *Enterococcus* spp., could be explained by the wide spectrum of polyphenols identified in the extract, which act as antimicrobial substances via different mechanisms of action (MOA). In fact, many MOA are ascribed to polyphenols, such as cytoplasmatic membrane damage, inhibition of nucleic acids, cell walls and cell membrane synthesis. Moreover, in addition to their direct antibacterial activity, a growing body of evidence suggests that polyphenols may interfere with some bacterial virulence factors such as enzymes, toxins and signal receptors [44].

The search for natural antimicrobial compounds is incited by the need to thwart the increasing bacterial resistance to antibiotics. In the biomedical field, this microbial antibiotic-resistance leads to a growing need for new, effective anti-infective materials for the prevention and delay of implant- and device-associated infections [53]. Anti-infective biomaterials have become a primary strategy to achieve this end. Natural polyphenols are interesting candidates for new antimicrobial agents to be loaded into or coated onto biomaterials. The development of nanoparticles carrying bioactive compounds with antimicrobial activity has been the target of investigations over the past years. In particular, several technologies have been developed at the nanoscale, such as nanoparticles, nanofibers, and nanocapsules, providing targeted delivery of polyphenols for therapeutic uses [54]. Recently, polyphenol delivery systems with antimicrobial activity have been described, focusing on nanoparticles based on chitosan as the main structural and functional material [55]. Polyphenols, ubiquitously expressed in plants have been shown to exert anticancer and immunomodulatory properties along with their anti-inflammatory and antimicrobial activities. However, some issues have been raised regarding the use of free polyphenols as medical drugs due to their fast metabolism and excretion in the human body. Indeed, this behavior might restrict or hamper their *in vivo* bioactive effects [56]. Therefore, a successful strategy for the use of *M. hallii* polyphenols as antimicrobial agents in the biomedical field may be require their administration in a form linked to or incorporated with nanomaterials able to support their controlled and prolonged release.

## 4. Materials and Methods

### 4.1. Chemicals and Materials

LC-MS grade methanol, acetonitrile and formic acid were purchased from Sigma-Aldrich (St. Louis, MO, USA). Millipore grade water was obtained with a Milli-Q water purification system (Millipore Corporation, Billerica, MA, USA). Filtration membranes (0.22 and 0.45 μm, cellulose acetate/cellulose nitrate mixed esters) were purchased from Millipore (Millipore Corporation). (−)-Epicatechin, (−)-catechin, (−)-epigallogatechin, (−)-gallocatechin and (−)-epigallocatechin-3-gallate were purchased by PhytoLab GmbH & Co.KG (Vestenbergsgreuth, Germany).

### 4.2. Plant Material

*M. hallii* was collected in July 2013 at Quito, Pichincha Province, Ecuador. Leaves were isolated manually from aerial parts and dried at room temperature for 3 weeks. Desiccated specimens were identified as *M. hallii* (O. Berg) McVaugh (syn. *Eugenia halli* Berg, *Amyrsia halli* (Berg) Kausel). Sample specimens have been deposited at the Herbarium of the University of Pavia (Department of Earth and Environmental Sciences, University of Pavia, Pavia, Italy) for future reference with the code: Quito (Pichincha, Ecuador), 26/07/2013, F. Bracco (PAV).

### 4.3. Extraction and Dialysis

Two 10 g aliquots of MHE were separately taken from the dried and finely ground leaves of *M. hallii* in 100 mL of H_2_O/methanol solution (70:30; % *v*/*v*) containing formic acid (0.1%, *v*/*v*), prepared in the dark, under constant stirring at room temperature for 24 h. Both extracts were filtered on a paper filter, gathered and freeze-dried. The residue was weighted and then reconstituted to 20 mL with Millipore-grade water and subdivided into two 10 mL aliquots. The first one was subjected to microbiological assays, the second was submitted to dialysis. Dialysis was performed using a Spectra/Por^®^ Biotech Regenerated Cellulose membrane (Spectrum Europe B.V., Breda, The Netherlands) with a nominal molecular weight cut-off (MWCO) of 3500 Da. An aliquot (10 mL) of MHE was submitted to dialysis in 1000 mL of Millipore-grade water for 24 h at 4 °C under constant stirring. The dialysate (low molecular weight fraction, LMW) was freeze-dried and the dry residue was assessed and dissolved in 10 mL of Millipore-grade water and subjected to microbiological assays and to UHPLC-PDA-hESI-MSn analysis, following filtration through 0.45 and 0.22 μm filters.

### 4.4. UHPLC-PDA-hESI-MSn Analysis

The experiment was performed using a Jasco X-LC system (Jasco, Easton, MD, USA) equipped with a quaternary pump, an UHPLC photodiode array detector (PDA) and a linear ion trap mass spectrometer LTQ-XL (Thermo Scientific, Waltham, MA, USA) through an h-ESI source. Separation was achieved on a Purospher^®^ STAR RP-18e (5 μm) LiChroCART^®^ 250-4 (250 × 4 mm^2^ i.d., 5 μm) with its corresponding guard column (both from Merck KGaA, Darmstadt, Germany). The mobile phase consisted of A (0.1% formic acid in water) and B (acetonitrile) at a flow rate of 1 mL/min with an injection volume of 5 μL. Gradient elution was carried out using the following timetable: 98% A/2% B 0–5 min, 60% A/40% B 5–40 min, 0% A/100% B 40–45 min, 0% A/100% B 45–47 min, 98% A/2% B 47–52 min, 98% A/2% B 52–57 min. The resulting total run time was 57 min, including column reconditioning. The sample tray was set at 4 °C and the column oven temperature was set at 24 °C. The chromatograms were recorded at λ 280, 220, 366 and 520 nm); spectral data were acquired in the range of 200–650 nm for all peaks. The ion trap operated in data dependent, full scan (80–1500 *m*/*z*), zoom scan and MS^n^ mode. To obtain the fragment ions a collision energy of 35% and an isolation of 2 *m*/*z* were applied; the voltage was kept at 3 kV for negative ionization and 5 kV for the positive one, the temperature of the capillary tube was 275 °C with a sheath gas flow rate of 45 arbitrary units and an auxiliary gas flow rate of 20 arbitrary units, while the ionization chamber was maintained at 100 °C. ThermoFisher Scientific Excalibur 2.0 software (SR2, Thermo Electron Corporation 1998–2006, Waltham, MA, USA) was used for data acquisition and processing. Three independent assays were performed to analyze the sample (filtered through a cellulose acetate/cellulose nitrate mixed esters membrane, 0.22 μm) and no relevant variations attributable to the nature of the detected fragments or their relative intensities were observed.

### 4.5. Bacterial Strains and Growth Conditions

The bacteria were recent clinical isolates, belonging to the Institute of Microbiology (University of Genova) collection. They comprised of: (i) ten *S. aureus* strains, including five methicillin-resistant (MRSA) and five methicillin-susceptible (MSSA) strains. Of the MRSA strains, three were multi-resistant (resistant to at least three classes of antibiotics); (ii) ten multi-resistant *Escherichia coli* strains; (iii) ten *Pseudomonas aeruginosa* strains; (iv) ten vancomycin-resistant and susceptible *Enterococcus faecalis* and *Enterococcus faecium* strains; and (v) ten group A streptococci (*Streptococcus pyogenes*) strains, which remain universally susceptible to penicillin. All isolates were identified at the species level using clinical methods and an API STAPH, API20E, API NE and API STREP system (bioMèrieux, Marcy l’Etoile, France) for *S. aureus*, *E. coli*, *P. aeruginosa*, *Enterococcus* spp. and *S. pyogenes* respectively. The antibiotype was determined using the disk diffusion test, as according to the latest Clinical and Laboratory Standards Institute (CLSI) guidelines [57]. Strains were cultured in Mueller-Hinton Broth, Mueller-Hinton agar, MacConkey agar and Columbia blood agar (Biolife, Milan, Italy) at 37 °C.

### 4.6. Susceptibility (MIC Determination)

The Minimum Inhibitory Concentration (MIC) of plant extract was determined using the broth microdilution method, following the CLSI guidelines [58]. In brief, exponentially growing bacteria (5 × 10^5^ cells per mL, final inoculum) were added to the various concentrations of plant extract, 2-fold serially diluted in 96-well microtitre plates of Mueller-Hinton broth or cation-adjusted Mueller Hinton broth with 5% of lysed horse blood for *S. pyogenes*. The following concentrations (gr/mL) of extracts were used: 0.5, 0.25, 0.125, 0.006, 0.031, 0.015, 0.007, 0.0039, 0.019, 0.00097, 0.00048. After 18–24 h of incubation at 37 °C, the concentration at which the plant extract prevented visible bacterial growth was identified as the MIC. All tests were performed in triplicate and executed three times. *S. aureus* ATCC 29213, *E. coli* ATCC 25922 and *P. aeruginosa* ATCC 27853 were added as control strains.

## 5. Conclusions

In conclusion, this study represents the first attempt to phytochemically characterize of *M. hallii* leaf extract and demonstrate its antimicrobial activity against drug resistant bacteria. The extract exhibits antimicrobial activity against *S. aureus*, *P. aeruginosa*, *S. pyogenes*, and *Enterococcus* spp. strains due to the presence of many different classes of polyphenolic compounds that possess antibacterial activity. The data reported in this paper reveal that *M. hallii* is a potential source of polyphenols with antimicrobial properties and shows the great potential of this species, not just for pharmaceutical applications, but also for biomedical, food and cosmetic applications.

## Figures and Tables

**Table 1:** MS and MS^n^ data of the compounds identified in LMW fraction of MHE.

Peak	RT (min)	*m*/*z*	HPLC-ESI-MS^n^ *m*/*z* (% of Base Peak)	Compound
1	2.43	191	173 (100), 127 (100)	Quinic acid
2	3.10	133	115 (100)	Malic acid
3	5.95	481	301 (100)	Hexahydroxydiphenoyl-glucose
4	11.03	169	125 (100)	Gallic acid
5	11.62	633	301 (100), 481 (5), 229 (5), 615 (2), 421 (5)	Hexahydroxydiphenoyl-galloylglucose
6	13.44	343	169 (100), 125 (10)	Monogalloyl-quinic acid
7	13.55	881	729 (20), 577 (30), 289 (5), 711 (10)	Procyanidin digallate
8	14.1	611 ^+^	449 (100), 287 (10)	Cyanidin-dihexoside
9	15.65	305	179 (100), 261 (45), 221 (85), 219 (80), 165 (25), 167 (30)	Gallocatechin
10	16.73	595 ^+^	443 (100), 287 (15)	Cyanidin-3-*O*-rutinoside
11	18.48	881	729 (20), 577 (30), 289 (2), 711 (10)	Procyanidin digallate
12	18.65	577	425 (100), 407 (40), 289 (20)	Procyanidin dimer
13	19.07	305	261 (50), 221 (90), 219 (80), 179 (100), 165 (30), 167 (10)	Epigallocatechin
14	19.22	577	425 (100), 407 (40), 289 (20)	Procyanidin dimer
15	20.49	289	245 (100), 205 (40), 203 (20), 137 (5)	Catechin
16	21.95	577	407 (40), 289 (15), 425 (100)	Procyanidin dimer
17	22.57	881	729 (20), 577 (30), 289 (2), 711 (10)	Procyanidin digallate
18	23.11	289	245 (100), 205 (35), 203 (20), 137 (5)	Epicatechin
19	23.66	457	169 (100), 305 (35)	Epigallocatechin gallate
20	24.99	729	577 (85), 289 (25)	Procyanidin-gallate
21	25.26	479	316 (100), 317 (90)	Myricetin 3-*O*-galactoside or Myricetin 3-*O*-glucoside
22	26.75	615	463 (100), 301 (10)	Quercetin hexosyl-gallate
23	26.93	449	316 (100), 317 (30)	Myricetin 3-*O*-arabinoside
24	27.43	463	316 (100), 317 (60)	Myricetin 3-*O*-rhamnoside
25	27.69	463	301 (100), 179 (2)	Quercetin 3-*O*-galactoside or Quercetin 3-*O*-glucoside
26	28.56	197^+^	179 (100), 135 (60)	Gluconic acid
27	28.83	433	301 (100), 179 (2)	Quercetin 3-*O*-arabinose
28	29.23	447	284 (100), 255 (10) 257 (5), 327 (25)	Kaempferol 3-*O*-glucoside
29	29.25	449 ^+^	287 (100)	Cyanidin-3-glucoside or Cyanidin-3-galattoside
30	30.23	447	301 (100), 179 (2)	Quercetin 3-*O*-rhamnoside
31	31.15	433	287 (40), 269 (100), 259 (10), 179 (5), 151 (2)	Aromadendrin-rhamnoside
32	31.43	505	316 (100), 463 (15)	Acylated myricitrin
33	32.55	483	331 (20), 169 (100)	Digalloylglucose
34	36.40	629	463 (85), 301 (100), 445 (10)	Cypellogin A or B
35	37.86	301	179 (100), 151 (50), 273 (20)	Quercetin
36	37.45	635	483 (100), 465 (5)	Trigalloylglucose
37	42.89	431	269 (100)	Apigenin-hexoside
38	43.72	343 ^+^	325 (10), 301 (2), 240 (100)	Pinobanksin 3-*O*-butyrate

^+^ Compounds revealed with positive ionization.

**Table 2:** *In vitro* antibacterial cumulative activity of MHE against ten *S. aureus*, ten *E. coli*, ten *P. aeruginosa*, ten *Enterococcus* spp., and ten *S. pyogenes* strains.

Strains (Number)	MIC Range (gr/mL)	50%	90%
*S. aureus* (10)	0.007–0.0019	0.0039	0.0039
*S. pyogenes* (10)	0.007–0.0039	0.0039	0.007
*Enterococcus* spp. (10)	0.0039–0.0019	0.0039	0.0039
*E. coli* (10)	0.25–>0.5	>0.5	>0.5
*P. aeruginosa* (10)	0.125–0.062	0.062	0.062

**Table 3:** MIC (mg/mL) of MHE against ten *S. aureus*, ten *S. pyogenes*, five *E. faecalis*, five *E. faecium*, ten *P. aeruginosa*, and ten *E. coli* strains. Frequencies (%) of the different bacterial species within MIC classes are shown.

Bacterial Species	Number of Strains	MIC mg/mL 1.90	MIC mg/mL 3.90	MIC mg/mL 7.00	MIC mg/mL 62.0	MIC mg/mL 125	MIC mg/mL 250	MIC mg/mL 510
**Gram-positive bacteria: frequencies (%)**								
*S. aureus*	10	10	80	10	-	-	-	-
*S. pyogenes*	10	-	60	40	-	-	-	-
*E. faecalis*	5	80	20	-	-	-	-	-
*E. faecium*	5	-	100	-	-	-	-	-
**Gram-negative bacteria: frequencies (%)**								
*P. aeruginosa*	10	-	-	-	90	10	-	-
*E. coli*	10	-	-	-	-	-	30	70

## Abbreviations

The following abbreviations are used in this manuscript:

MHE	*Myrcianthes hallii* extract
LMW	low molecular weight
MRSA	methicillin-resistant *Staphylococcus aureus*
MSSA	methicillin-susceptible *Staphylococcus aureus*
CLSI	Clinical and Laboratory Standards Institute
MIC	Minimum inhibitory concentration
MOA	mechanism of action
